# Influenza A viruses escape from MxA restriction at the expense of efficient nuclear vRNP import

**DOI:** 10.1038/srep23138

**Published:** 2016-03-18

**Authors:** Veronika Götz, Linda Magar, Dominik Dornfeld, Sebastian Giese, Anne Pohlmann, Dirk Höper, Byung-Whi Kong, David A. Jans, Martin Beer, Otto Haller, Martin Schwemmle

**Affiliations:** 1 Institute of Virology, University Medical Center Freiburg, D-79104 Freiburg, Germany; 2 Institute of Diagnostic Virology, Friedrich-Loeffler-Institute, 17493 Greifswald-Insel Riems, Germany; 3 Center of Excellence for Poultry Science, University of Arkansas, Fayetteville, AR 72701, USA; 4 Nuclear Signaling Laboratory, Department of Biochemistry and Molecular Biology, Monash University, Melbourne, Victoria, Australia

## Abstract

To establish a new lineage in the human population, avian influenza A viruses (AIV) must overcome the intracellular restriction factor MxA. Partial escape from MxA restriction can be achieved when the viral nucleoprotein (NP) acquires the critical human-adaptive amino acid residues 100I/V, 283P, and 313Y. Here, we show that introduction of these three residues into the NP of an avian H5N1 virus renders it genetically unstable, resulting in viruses harboring additional single mutations, including G16D. These substitutions restored genetic stability yet again yielded viruses with varying degrees of attenuation in mammalian and avian cells. Additionally, most of the mutant viruses lost the capacity to escape MxA restriction, with the exception of the G16D virus. We show that MxA escape is linked to attenuation by demonstrating that the three substitutions promoting MxA escape disturbed intracellular trafficking of incoming viral ribonucleoprotein complexes (vRNPs), thereby resulting in impaired nuclear import, and that the additional acquired mutations only partially compensate for this import block. We conclude that for adaptation to the human host, AIV must not only overcome MxA restriction but also an associated block in nuclear vRNP import. This inherent difficulty may partially explain the frequent failure of AIV to become pandemic.

Avian influenza A viruses (AIV) are transmitted occasionally from their natural aquatic bird reservoir to humans. Most of these transmissions are self-limiting except in very rare cases in which a stable virus lineage emerges in the new host due to adaptation^1^,^2^. In particular, zoonotic transmissions of AIVs, including the H5N1 subtype, pose a constant threat for the human population. In 1997, the first human infection with a highly pathogenic avian influenza virus of the H5N1 subtype was reported from Hong Kong^3^. Since then the virus has spread from Asia to Europe and Africa, circulating in wild birds and poultry. As of September 2015, 844 human infections from different countries have been laboratory-confirmed^4^. Of these, 449 were fatal, resulting in a mortality rate of about 53%. Most patients were in close contact to poultry prior to infection, indicating a direct transmission from birds to humans. Although sustained human-to-human transmission has not been observed, the emergence of human-adapted and transmissible influenza A viruses of the H5N1 or H7N9 subtype is a major concern since years.

To allow efficient infection of mammals including humans and subsequent human-to-human transmission, adaptive changes must occur in nearly all AIV viral proteins^5^,^6^,^7^. Of note, a number of mutations must be acquired which favor airborne transmission necessary for efficient spread^1^,^8^. Amino acid substitutions in the hemagglutinin (HA) surface glycoprotein are required to switch host cell receptor binding specificity from avian α2,3-linked to human α2,6-linked sialic acids^9^,^10^,^11^. Passaging experiments in ferrets recently revealed that H5N1 viruses can acquire mutations in HA that allow airborne transmission in this animal model^10^,^11^. Likewise, mutations in the viral polymerase subunits, such as E627K in PB2, are necessary to increase the avian polymerase activity that is otherwise poor in human cells^6^,^12^,^13^. Of note, the recently circulating H5N1 strains in Egypt, derived from the Qinghai Lake viruses^14^, maintained the major adaptive mutation E627K in PB2 as well as some of the mutations in HA known to be required for airborne transmission, potentially increasing the pandemic potential of these viruses^15^.

However, despite extensive research efforts, the evolutionary forces driving adaptation to the human host are still not completely understood, and thus the real pandemic potential of AIV, including H5N1 viruses is difficult to estimate. In particular, the host innate immune system exerts a strong selective pressure against new viruses attempting to invade the mammalian host^16^,^17^,^18^,^19^, yet surprisingly little is known about adaptive mutations that counteract cellular restriction factors of the innate immune system. Interferons (IFNs) inhibit invading influenza A viruses at early stages of infection by virtue of a number of induced antiviral factors, including Mx proteins and IFITM3^20^. Mx proteins are interferon-induced, dynamin-like large GTPases^21^. They are encoded by two paralogous genes at the Mx locus on human chromosome 21. Myxovirus resistance gene1 (*MX1*) encodes the human MxA protein while *MX2* encodes the related MxB protein exhibiting 63% amino acid identity. Both proteins are highly induced by type I (alpha/beta) or type III (lambda) IFN but display different antiviral specificities. While MxB restricts HIV-1 and other primate lentiviruses by preventing nuclear import^21^,^22^,^23^,^24^,^25^, human MxA has a broad antiviral activity against many RNA viruses, including influenza A viruses, and DNA viruses^21^. Mx proteins are highly conserved in vertebrates but display different antiviral specificities. Significantly, all avian Mx proteins analyzed to date have been antivirally inactive against influenza A viruses^26^,^27^. Available evidence indicates that MxA inhibits influenza A viruses at two early steps in the life cycle, interfering with both the transcription of viral RNA of incoming vRNPs (primary transcription) and the amplification of vRNA from cRNA copies (secondary transcription), but the exact mode of action is presently not known^21^,^28^. Primary transcription is most likely prevented via retention of incoming vRNPs in the cytoplasm with the help of as yet unknown IFN-induced cellular factor(s)^29^. Secondary transcription appears to be blocked by cytoplasmic sequestering of newly synthesized NP and PB2^30^,^31^. Interestingly, human MxA was found to be more active against influenza A viruses of avian origin, including H5N1 viruses, than against seasonal and pandemic human strains^32^. Importantly, NP was identified as the main viral factor determining the strength of MxA inhibition^30^. We recently mapped a specific area of NP of the 1918 pandemic H1N1 virus that largely governs MxA escape^33^. Three residues, 100I/V, 283P, and 313Y, were sufficient to mediate MxA evasion. These surface-exposed amino acids are located in a distinct area of the body domain of NP and are nearly 100% conserved in all 1918-descendant human circulating viruses, including the seasonal H3N2 viruses of today. Of note, the mutations are virtually absent in the NPs of avian circulating viruses including AIV of the H5N1 subtype. The residues responsible for MxA escape of the 2009 pandemic H1N1 virus were found in a comparable cluster in NP, consisting of amino acid 53D, 100 V and 313 V. We could recently provide preliminary evidence that artificial introduction of the MxA escape cluster in AIV of the H5N1 subtype might be accompanied by reduced viral fitness^33^. To determine whether H5N1 viruses have the potential to acquire MxA resistance without fitness loss we generated and analyzed a panel of H5N1 viruses with the MxA escape cluster of the 1918 NP. We found that introduction of the major escape mutations in NP consistently resulted in viruses with severe growth defects, which rapidly acquired additional secondary NP mutations that genetically stabilized the virus and contributed to viral fitness. We further show that the severe growth defects mediated by the MxA escape cluster were caused by a defective transport of vRNPs into the cell nucleus, which was partially compensated by the stabilizing mutations. Unexpectedly, most stabilizing NP mutations caused a loss of MxA escape and restored MxA sensitivity to the level of wild-type virus. These findings suggest that it is an extreme challenge for AIVs to escape MxA while maintaining viral fitness.

## Results

### Amino acid changes in NP required for MxA escape result in reduced viral fitness of avian H5N1 and H7N7 viruses

**Figure 1:**
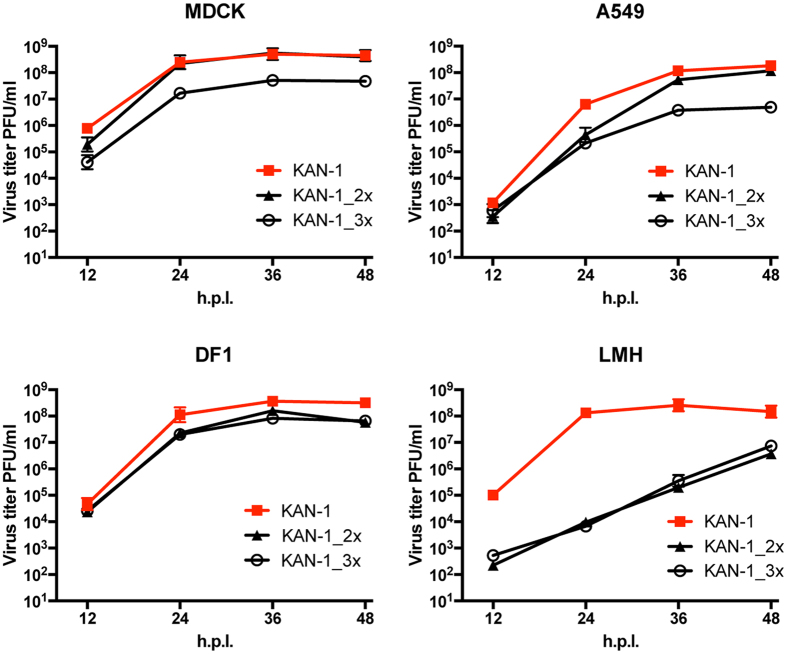
Recombinant KAN-1 viruses carrying MxA escape mutations show a degree of attenuation in different cell culture systems. Cells were infected at an MOI of 0.001 with wild-type KAN-1 (KAN-1) or the indicated mutant viruses. At the indicated hours post infection (h.p.i.), virus titers were determined by plaque assay. Error bars indicate the standard deviation of the mean of at least three independent experiments.

We recently described an amino acid patch in the nucleoprotein of the prototypic strain A/Brevig Mission/1/1918(H1N1), consisting of 100I/V, 283P, 313Y that confers resistance against MxA. To investigate the impact of these human-adaptive amino acids on an AIV, we introduced either two (R100I, F313Y) or all three (R100V, L283P, F313Y) amino acids^33^ into the NP of the avian strain A/Thailand/1(KAN-1)/2004(H5N1)^34^,^35^. The resulting mutant viruses were designated KAN-1_2x or KAN-1_3x, respectively. Parental wild-type KAN-1 and mutant KAN-1_2x were rescued efficiently and produced virus stocks with high titers (>10^7^ PFU/ml), as shown previously^33^. Apart from MDCK cells, KAN-1_2x grew to slightly lower titers than wild-type KAN-1 in all cell lines tested, including human A549 cells and avian LMH and DF1 cells. In contrast, independent rescues of KAN-1_3x resulted in virus stocks with either comparatively low titers (<10^4^ PFU/ml) and no additional mutations in NP, or in a virus stock with high titers (>10^7^ PFU/ml) and one of several various additional mutations in NP (Table S1). KAN-1_3x without additional mutations (e.g. virus stock #1; Table S1) exhibited a pronounced growth defect compared to wild-type KAN-1 in all cell lines. However, relatively high viral titers of >10^6^ PFU/ml were observed in all cases at 48 h post infection (Fig. 1). Of note, both KAN_2x and KAN_3x viruses were most attenuated in LMH cells, where virus titers of both viruses were reduced by more than 4 log10 from wild-type KAN-1 (Fig. 1).

To test whether virus attenuation is a general feature of MxA escape, we introduced two (100I, 313Y) or all three (100 V, 283P, 313Y) of the NP amino acids associated with MxA escape into the mouse-adapted SC35M (H7N7) virus which lacks all of the important amino acid substitutions known to contribute to MxA resistance^33^,^36^. While rescue of SC35M_3x failed, recombinant SC35M_2x could be readily generated. It was found to be mildly attenuated in MDCK, A549 and DF1 cells but severely attenuated in LMH cells, in agreement with the KAN-1 results shown above (supplementary Figure S1).

It was conceivable that the attenuated phenotype observed was due to diminished viral polymerase activity in the context of the mutated NPs. We therefore reconstituted the viral polymerases of either KAN-1 or SC35M in the presence of wild-type or mutant NPs in cell lines without antivirally-active MxA. In all cases, polymerase activity was comparable between wild-type and mutant NPs irrespective of the cell line (supplementary Figures S2A,B and S3).

These findings demonstrate that introduction of MxA escape mutations into the NP of avian influenza A viruses results in impaired viral growth in mammalian and avian cells, but does not affect polymerase activity.

### MxA escape mutants acquire additional adaptive mutations in NP

To screen for possible mutations in NP, samples from growth kinetics shown in Fig. 1 were subjected to sequencing, including kinetics from A549 cells constitutively expressing MxA (A549-MxA)^29^. A549-MxA, MDCK, DF-1, and LMH cells were infected with either KAN-1_2x (virus stock #1) or KAN-1_3x (virus stock #1 or #2). Both KAN-1_3x virus stocks had low virus titers (<10^4^ PFU/ml) and no detectable additional mutations in NP (supplementary Table S1). After 48 h of infection, total RNA was extracted and screened for the emergence of additional mutations in NP by sequencing. No new mutations were found in KAN-1_2x progeny. In contrast, various single amino acid mutations were detected in the NP of all KAN-1_3x progeny (supplementary Table S1). Depending on the cell type, different NP mutations were found. Mutation G16D occurred in A549-MxA as well as in MDCK cells, whereas Y385C was regularly found in all cell lines. Mutation D101Y was only found in LMH cells. No additional NP mutations were detected in the virus stocks used for infection and the originally introduced amino acid substitutions conferring MxA escape were maintained in all cases.

We next analyzed the full-genome sequences of KAN-1_3x from infected A549-MxA, MDCK, or LMH cells (supplementary Table S2). Deep sequencing confirmed the observed additional mutations in NP and showed no further mutations in NP. The viruses passaged from KAN-1_3x stock #1 all harbored an additional mutation in PB1 (L10V). Since L10V was already present in the low titer virus stock #1 used for infection (supplementary Table S2) its contribution to viral growth is probably of minor importance. Consistently, infection of MDCK cells with plaque-purified KAN-1_3x + 16D virus harboring in addition the PB1 mutation L10V (KAN-1_3x + 16D + 10 V) revealed no differences in viral growth compared to recombinant KAN-1_3x + 16D (data not shown). Moreover, the stock consisted of a mixture of viruses with mutations in PB2 and NA at low frequencies, which appeared to be lost during further passaging. However, a new mixture of viruses was found in LMH cells featuring additional mutations in HA. Similarly, KAN-1_3x with the additional NP mutation Y385C (that originated from virus stock #2) proved to be a mixture of viruses with 4 different mutations in PB2. Infection of LMH cells with stock #2 resulted in viruses with D101Y as the prevalent mutation in NP (72%) and Y385C at low frequency (22%), while the 4 mutations in PB2 were maintained (supplementary Table S2).

### Most adaptive mutations in NP restore MxA sensitivity to KAN-1 wild-type levels

**Figure 2:**
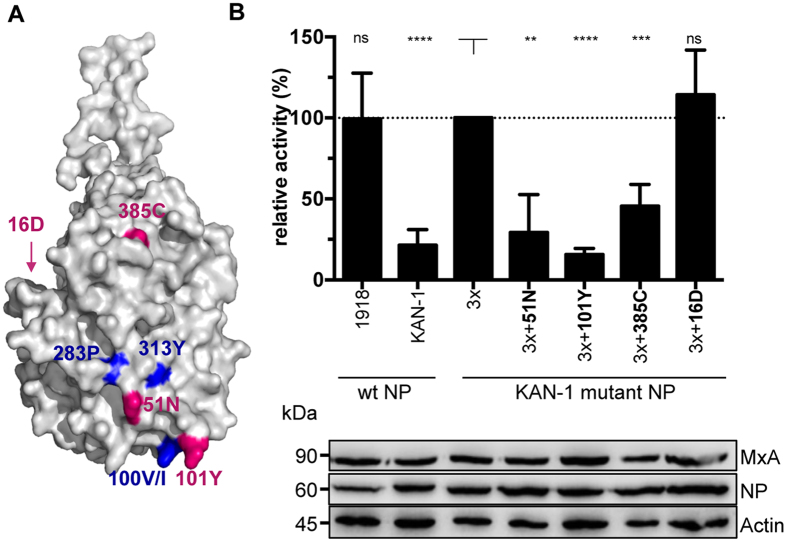
Additional adaptive mutations in NP affect viral polymerase activity in the presence of MxA. (**A**) Structural model of an H5N1 virus nucleoprotein. Major amino acids allowing MxA escape are highlighted in blue. Additional mutations acquired during propagation of KAN-1_3x in tissue culture are marked in magenta. The program PyMOL was used to assign the indicated positions based on the structural model of A/HK/483/97(H5N1) NP (PDBcode: 2Q06). (**B**) Polymerase activity in the presence of MxA. HEK293T cells were transiently transfected with expression plasmids coding for PB2, PB1, PA of KAN-1, the indicated NP proteins, a minigenome encoding the firefly luciferase and a renilla luciferase expression plasmid to normalize for variations in expression efficiency. Polymerase activity in the presence of antivirally inactive MxA_T103A was used to normalize the data obtained with MxA (relative activity). Western blot analysis was performed to determine the expression levels of NP and MxA. Error bars indicate the standard deviation of the mean of at least 3 independent experiments. Student’s T test was performed to determine the P value **P < 0.01, ***P < 0.001, ****P < 0.0001, not significant (ns).

Most of the additional NP mutations are located on the surface of the NP body domain close to the known MxA escape patch (Fig. 2A). 51N is within the center of the patch, and 101Y is adjacent to the escape-associated amino acid 100 V. The other two amino acids, 385C and 16D, are somewhat more distant from the main escape cluster but also reside to areas known to influence MxA resistance^33^. 16D resides in the flexible N terminus that is not present in the crystal structure (Fig. 2A).

To determine the effect of these adaptive mutations on the activity of the viral polymerase of KAN-1 in relation to MxA, polymerase reconstitution assays were performed in the presence of active or inactive MxA. In the presence of inactive MxA_T103A, none of the mutations had a strong positive or negative effect on the viral polymerase in 293T cells (supplementary Figure S2A), indicating that the function of the polymerase was unaffected. Similar results were observed in LMH cells (supplementary Figure S3). In the presence of active MxA, however, three of the acquired mutations (51N, 101Y, 385C) diminished the KAN-1_3x polymerase activity to the level of wild-type KAN-1 (Fig. 2B). In contrast, substitution G16D maintained the MxA escape phenotype of KAN-1_3x (Fig. 2B). Interestingly, an aspartic acid (D) at position 16 had previously been described to contribute to MxA escape of A/Brevig Mission/1/1918(H1N1)^33^. To verify these findings in a different genetic background, the additional KAN-1 mutations in NP (101Y, 385C and 16D) were introduced into SC35M_3x and tested in the polymerase reconstitution assay. The results were much in agreement with those for KAN-1 (supplementary Figure S2B, S2C).

In conclusion, additional mutations in NP and other viral proteins are acquired upon virus propagation. Most additional NP mutations restore sensitivity to the antiviral action of MxA with the exception of G16D, as revealed in polymerase reconstitution assays.

### Adaptive mutations G16D and Y385C in NP are not able to completely compensate the viral fitness loss caused by MxA escape mutations in KAN-1

**Figure 3:**
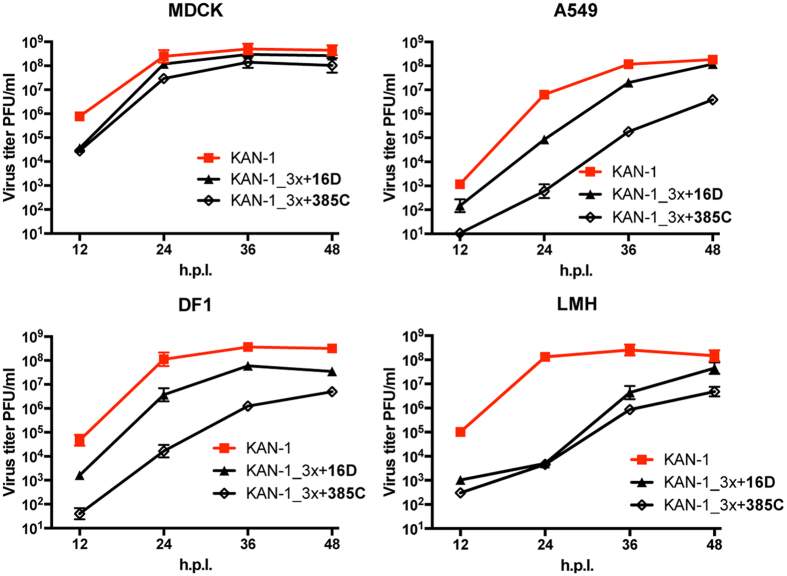
The additional mutations in NP of KAN-1_3x only partially restore viral fitness. Cells were infected at an MOI of 0.001 of wild-type KAN-1 (KAN-1) or the indicated mutant viruses. At the indicated hours post infection (h.p.i.), virus titers were determined by plaque assay. Error bars indicate the standard deviation of the mean of at least three independent experiments.

Recombinant KAN-1_3x viruses harboring either NP mutation G16D (KAN-1_3x + 16D) or Y385C (KAN-1_3x + 385C) were generated. Virus stocks were obtained at high titers (>10^7^ PFU/ml) that did not contain any additional mutations in NP (data not shown). KAN-1_3x + 16D replicated more efficiently than KAN-1_3x + 385C in MDCK, A549, and DF1 cells, but both viruses grew generally to lower titers than wild-type KAN-1 in all cell lines tested (Fig. 3). Viral growth curves of these mutant KAN-1_3x viruses did not differ greatly from those of the parental KAN-1_3x viruses (low virus stock #1) that had acquired adaptive mutations in NP (compare Figs 1 and 3). In particular, attenuation of KAN-1_3x was most pronounced in LMH cells (Fig. 1), as it was for both mutant viruses, showing >4 log 10 reduced viral titers in comparison to wild-type virus (Fig. 3). Sequencing of KAN-1_3x + 16D and KAN-1_3x + 385C released 48 h post infection from MDCK, A549, DF1 and LMH cells revealed no additional mutations in NP, suggesting that compensatory mutations that might enhance viral fitness are not readily acquired.

Although we repeatedly failed to recue SC35M_3x, the generation of recombinant SC35M_3x + 385C and SC35M_3x + 16D was possible and resulted in virus stock titers of >10^7^ PFU/ml and no further mutation in NP, highlighting the stabilizing feature of both 16D and 385C. Similar to the KAN-1 mutants, SC35M_3x + 385C growth was impaired in all cell lines investigated compared to wild-type SC35M (supplementary Figure S5A), while SC35M_3x + 16D replicated as efficiently as wild-type SC35M in MDCK and A549 (supplementary Figure S5A). This unimpaired growth in mammalian cell lines is possibly due to numerous mammalian-adaptive mutations present in various viral proteins of the mouse-adapted SC35M strain^36^. Together, these results show that the stabilizing mutations G16D and Y385C are sufficient to partially overcome the growth deficit mediated by the MxA escape mutations. However, these stabilizing mutations do not completely rescue the attenuation phenotype in an AIV of the H5N1 subtype in avian and mammalian cells, whereas especially the stabilizing mutation G16D restored viral growth of a mouse-adapted SC35M strain to wild-type levels in mammalian cells. Of note, the mutation G16D alone had no effect on SC35M growth in MDCK cells (data not shown).

### Amino acid substitution G16D in NP does not interfere with MxA escape

**Figure 4:**
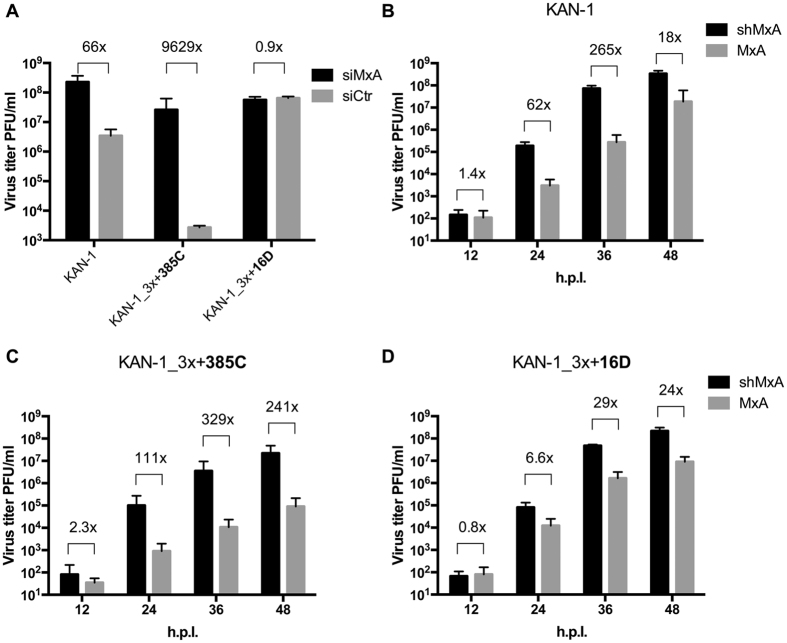
MxA escape of KAN-1_3x is influenced by the additional mutations in NP. (**A**) A549-MxA cells were treated with siRNAs targeting MxA (siMxA) or a non-targeting siRNA (siCtr). Three days post transfection, cells were infected with wild-type KAN-1 (KAN-1) or the indicated mutant viruses at a MOI of 0.001 and virus titers were determined by plaque assay 48 hours post infection. Error bars indicate the standard deviation from the mean of 3 independent experiments. Fold differences in virus titers are indicated. (**B**–**D**) A459 cells expressing (MxA) or lacking MxA (shMxA) were infected at an MOI of 0.001 of wild-type KAN-1 (KAN-1) or the indicated mutant viruses. At the indicated hours post infection (h.p.i.), virus titers were determined by plaque assay. Error bars indicate the standard deviation of the mean of 3 independent experiments. Fold differences in virus titers are indicated.

Acquisition of amino acid substitution G16D in NP did not interfere with the effect of the MxA escape mutations in polymerase reconstitution assays (Fig. 2B). We next evaluated whether unimpaired MxA escape could also be demonstrated in the context of a viral infection. A549-MxA cells that constitutively express MxA were transfected with an siRNA targeting MxA (siMxA) or a non-targeting siRNA (siCtrl) and incubated for 72 h. Efficient knockdown of MxA expression was confirmed by Western blot (supplementary Figure S4). The silenced and control cells were infected with wild-type KAN-1, mutant KAN-1_3x + 385C, or mutant KAN-1_3x + 16D. Virus titers of wild-type KAN-1 and KAN-1_3x + 385C were significantly lower in MxA-expressing cells than in MxA-deficient cells. The reduction in virus titers 48 hours post infection was 66- and 9629-fold, respectively, demonstrating that both viruses were restricted by MxA (Fig. 4A). In contrast, viral titers in KAN-1_3x + 16D infected cells were unaffected by the presence or absence of the restriction factor, indicating unimpaired MxA escape (Fig. 4A). Comparable results were obtained when cells constitutively expressing MxA (A549-MxA) or expressing an MxA-specific shRNA (A549-shMxA)^29^ were infected. At 36 hours post infection, virus titers of both wild-type KAN-1 and KAN-1_3x + 385C were considerably lower in A549-MxA cells than in A549-shMxA cells by 265- and 329-fold, respectively (Fig. 4B,C). The difference in titers of KAN-1_3x + 16D 36 h after infection was barely 29-fold, indicating unimpaired MxA escape (Fig. 4D). As expected we could recapitulate the differences in MxA sensitivity for the SC35M recombinant viruses (supplementary Figure S5B–D). Similar to KAN-1_3x + 16D, SC35M_3x + 16D showed unimpaired MxA escape, whereas wild-type SC35M and SC35M_3x + 385C remained MxA sensitive.

### Attenuation of MxA escape viruses is associated with inefficient nuclear import of incoming vRNPs

**Figure 5:**
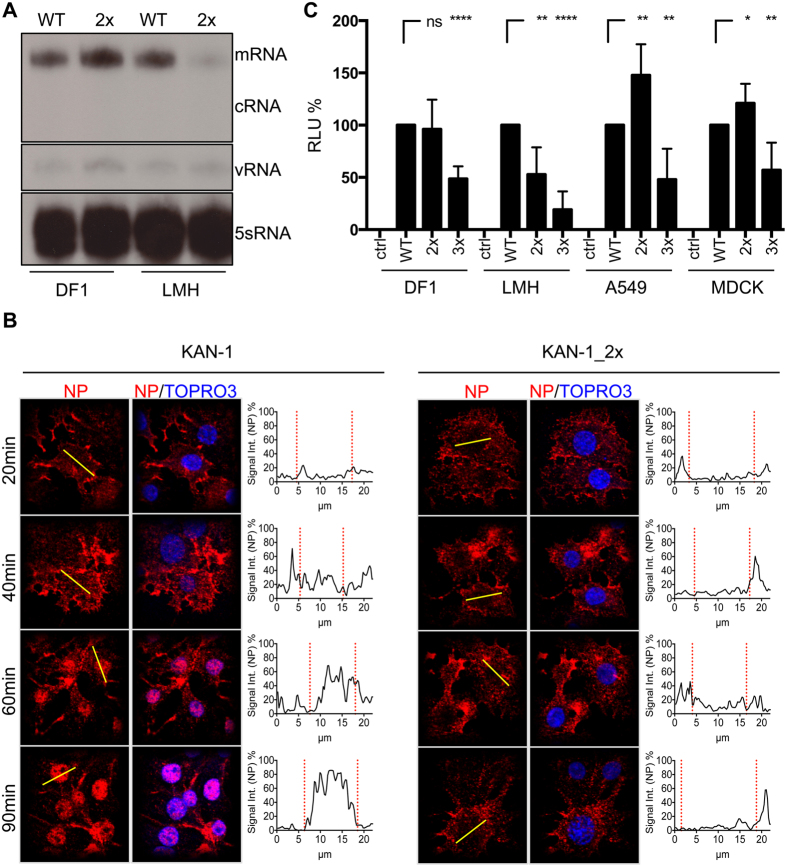
Attenuation due to MxA escape is caused by inefficient nuclear import of incoming vRNPs. (**A**) DF1 or LMH cells were infected with wild-type KAN-1 (WT) or KAN-1_2x (2x) at an MOI of 50 in the presence of 100 μg/ml cycloheximide and primary viral transcript levels were determined 3 hours later. Viral RNA species were detected by primer extension analysis using specific primers for the NA segment. (**B**) Detection of incoming vRNPs in LMH cells infected with wild-type KAN-1 (KAN-1) or KAN-1_2x at an MOI of 50 in the presence of 100 μg/ml cycloheximide. Infection was carried out on ice for 40 minutes to synchronize virus entry before incubation at 37 °C for the times indicated. Incoming vRNPs were visualized by NP staining and cell nuclei by TOPRO3 staining. Histograms indicate the NP fluorescence intensity along the yellow line in selected cells. Red dashed lines indicate the borders of the cell nuclei identified by TOPRO3 staining. (**C**) Equal amounts of virus like particles (VLP) (see supplementary Figure S7A), reconstituted with either wild-type (WT) or mutant NP proteins (2x, 3x) and a minigenome coding for a firefly luciferase, were used to infect the indicated cell lines. Luciferase activity was determined 10 h post infection. HA was omitted as a negative control (ctrl). Error bars indicate the standard deviation from the mean of at least 4 independent experiments. Student’s T test was performed to determine the P value *P < 0.05, **P < 0.01, ****P < 0.0001, not significant (ns).

To gain insights into the mechanism responsible for the attenuation associated with MxA escape, we investigated different steps in the viral replication cycle of KAN-1. Studies with KAN-1_3x are not possible due to the low yield of virus stocks lacking further mutations in NP (supplementary Table S1). We therefore made use of KAN-1_2x, which lacks any compensatory mutations in NP and yields high viral titers (supplementary Table S1). KAN-1_2x growth was most attenuated in LMH cells, and only slightly attenuated in DF1 and mammalian cells (Fig. 1). We first addressed the question of whether primary transcription was affected in KAN-1_2x infected LMH cells. In order to differentiate between primary and secondary transcription, the drug cycloheximide was used to block protein synthesis. In cycloheximide-treated cells, primary transcription occurs normally, while subsequent secondary transcription and replication steps relying on ongoing viral protein synthesis are blocked. DF1 and LMH cells were infected at a high MOI of 50 in the presence of the drug. Cells were lysed 3 h later and the accumulation of NA segment RNA transcripts was analyzed by primer extension (Fig. 5A). As expected, no cRNA was detected in any sample due to cycloheximide treatment of the cells^37^. In DF1 cells, comparable mRNA levels for wild-type KAN-1 (WT) and KAN-1_2x (2x) were observed, indicating proper transport of the incoming vRNPs into the cell nucleus and comparable primary transcription. In sharp contrast, mRNA levels of KAN-1_2x in LMH cells were considerably lower than those of wild-type KAN-1. Thus, either primary transcription of KAN-1_2x or another step prior to primary transcription, such as nuclear import, was defective in LMH cells in which the virus was severely attenuated.

To investigate nuclear import of incoming vRNPs, we monitored LMH cells infected with either wild-type KAN-1 or KAN-1_2x by confocal immunofluorescence microscopy. LMH cells were infected in the presence of cycloheximide at an MOI of 50. To synchronize virus entry, infection was performed on ice for 40 minutes before medium exchange and switch to 37 °C. After 20, 40, 60 and 90 minutes post infection, cells were fixed and stained for NP, the major component of vRNPs (Fig. 5B). In KAN-1-infected cells, nuclear accumulation of NP was observed as early as 60 minutes post infection. In contrast, the majority of the NP signal remained in the cytoplasm of cells infected with KAN-1_2x. Similar results were obtained when SC35M_2x was used for infection (supplementary Figure S6). These results indicate that the NP mutations associated with MxA escape lead to inefficient nuclear vRNP import and, as a consequence, to reduced primary transcription, explaining the severe attenuation observed in LMH cells.

As shown in supplementary Tables S1 and S2, the mutant virus KAN-1_3x is either genetically unstable or available only in very low virus stock titers (supplementary Table S1), impeding further mechanistic studies. In order to circumvent this difficulty and to investigate the effect of all three major MxA escape mutations on nuclear import, we generated KAN-1 virus-like particles (VLPs) containing a minigenome encoding a firefly luciferase. Such VLPs allowed us to examine the process of primary transcription in infected cells by measuring luciferase reporter activity (Fig. 5C). No significant differences in reporter activity were observed after infection of DF1 cells with comparable amounts of VLPs (supplementary Figure S7A) reconstituted in the presence of wild-type NP (NP_WT) or mutant NP_2x, as expected (Fig. 5C). In contrast, VLPs reconstituted with NP_2x had reduced reporter activity in LMH cells, approximately 50% compared to NP_WT, and reporter activity was even lower with NP_3x. Infection of A549 or MDCK cells with VLPs reconstituted with NP_2x revealed enhanced luciferase activity as compared to NP_WT, whereas reporter signal strength was again significantly lower with NP_3x (Fig. 5C). Next, we generated VLPs with NP_3x + 16D to determine whether the mutation G16D might improve nuclear vRNP import. With the exception of MDCK cells, infection of LMH, DF1 and A549 cells with VLPs reconstituted with NP_3x + 16D resulted in significantly higher levels of reporter activity compared to infections with VLPs reconstituted with NP_3x (supplementary Figure S7B). In contrast, infection of these cells with VLPs reconstituted with NP_3x + 385C revealed no statistical significant increase in reporter activity compared to infections with VLPs reconstituted with NP_3x (supplementary Figure S7B), suggesting that the mutation Y385C does not improve vRNP import.

NP_3x and NP_3x + 16D are as active or even more active than NP_WT in the polymerase reconstitution assay (supplementary Figures S2A and S3). We therefore assume that the differences in reporter activity observed with VLPs are indicative of differences in infection events preceding primary transcription, such as impaired nuclear vRNP import. The VLP data therefore support the findings that MxA escape mutations interfere with efficient nuclear vRNP import and indicate that the degree of interference depends on the host cell. They further show that NP mutation G16D partially restores vRNP nuclear import efficiency of KAN-1_3x in some cell types.

### MxA escape viruses undergo normal fusion and uncoating processes

To exclude the remote possibility that the very early steps of viral fusion and uncoating are affected and lead to the observed block in nuclear import of vRNPs harboring MxA escape mutations, we used labeled virus particles to infect LMH cells. As shown in supplementary Figure 6, SC35M_2x displayed the same block in nuclear import as KAN-1_2x. Therefore, and because SC35M is a BSL-2 pathogen in Germany, we made use of SC35M for these experiments. Wild-type SC35M and mutant SC35M_2x viruses were labeled with SP-DiOC18 and R18. The double-labeled non-fused viruses are red (R18), due to FRET and self-quenching of DiOC18. Upon fusion, a green signal appears. Both viruses showed comparable fusion efficiency in LMH cells (supplementary Figure S8A). To analyze the uncoating event in more detail, LMH cells were infected at an MOI of 50 with wild-type SC35M or mutant SC35M_2x virus in the presence of cycloheximide and cells were fixed and stained for NP as well as for the matrix protein M1 (supplementary Figure S8B). Most of the wild-type NP signal translocated into the nucleus within 90 minutes after infection, whereas NP of SC35M_2x remained in the cytoplasm. M1 showed cytoplasmic distributions in both cases, and no pronounced colocalization between NP and M1 was observed, indicating proper uncoating.

### Impaired vRNP import through importin-α/β in MxA escape viruses

Influenza A virus vRNPs are believed to rely principally on the importin-α/β nuclear import complex in order to enter the cell nucleus, where nuclear localization signals (NLSs) within NP are recognized by importin-α within the importin-α/β heterodimer^38^,^39^,^40^,^41^,^42^. Importin-β then mediates all subsequent steps of transport of the ternary vRNP-importin cargo complex across the nuclear pore embedded in the nuclear envelope, followed by release within the nucleus. However, it is known that nuclear transport can be mediated through non-importin-dependent mechanisms^43^, in some cases through switching between importin-dependent and -independent pathways according to cell or other signals^43^,^44^.

**Figure 6:**
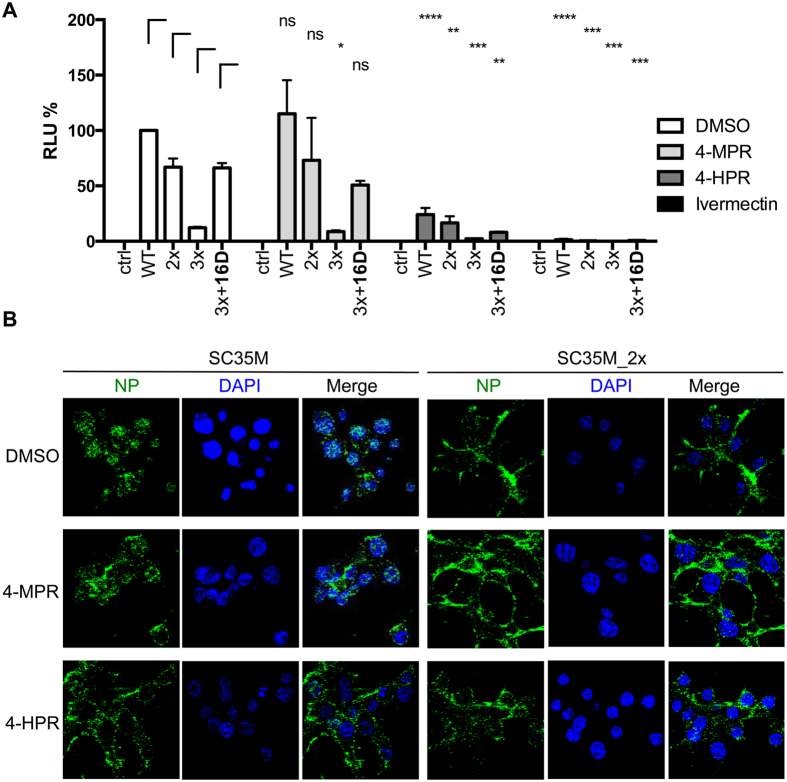
Nuclear import of vRNPs carrying MxA escape mutations depends on importin-α/β. (**A**) Equal amounts of KAN-1 VLP reconstituted with either wild-type (WT) or the indicated KAN-1 mutant NP proteins (2x; 3x; 3x + 16) and a minigenome coding for a firefly luciferase were used to infect LMH cells in the presence of no compound, 15 mM inactive N-(4-methoxyphenyl) (4-MPR) as a negative control, 15 μM N-(4-hydroxyphenyl) retinamide (4-HPR) or 10 μM ivermectin. Luciferase activity was determined 10 h post infection. HA was omitted as a negative control (ctrl). Error bars indicate the standard deviation from the mean of 2–4 independent experiments. Student’s T test was performed to determine the P value *P < 0.05, **P < 0.01, ***P < 0.001, ****P < 0.0001, not significant (ns). (**B**) Nuclear vRNP import in the presence of import inhibitors. LHM cells were pretreated with cycloheximide and infected with wild-type SC35M or mutant SC35M_2x at an MOI of 50 in the presence of 15 μM N-(4-hydroxyphenyl) retinamide (4-HPR) or 15 μM inactive N-(4-methoxyphenyl) (4-MPR) as a negative control. Infection was carried out on ice for 40 minutes to synchronize virus entry and further incubated at 37 °C. After 90 minutes, cells were fixed and incoming vRNPs were visualized using a NP-specific antibody. Cell nuclei were visualized by DAPI staining. Images were acquired using a Zeiss confocal laser microscope.

To assess whether nuclear import of incoming vRNP composed of NPs with or without MxA adaptive mutations is importin-α/β-dependent, we made use of the compounds ivermectin, an importin-α/β- specific nuclear import inhibitor^45^, known to inhibit nuclear import of various viral proteins^46^,^47^, and N-(4-hydroxyphenyl) retinamide (4-HPR), a specific inhibitor of Dengue virus nonstructural protein 5 importin-α/β-dependent nuclear import^48^ along with the 4-HPR related molecule N-(4-methoxyphenyl) retinamide (4-MPR) that lacks inhibitory activity^48^. LMH cells treated with the three compounds were infected with KAN-1 VLPs reconstituted with wild-type NP or the mutant NPs_2x, 3x or 3x + 16D (Fig. 6A). Reporter activity was comparable between non-treated cells and cells treated with the inactive compound 4-MPR for all VLP species, as expected, whereas cells treated with 4-HPR showed a general decrease in reporter activity for all VLP variants. Treatment with ivermectin completely abrogated nuclear import of all different vRNPs resulting in no detectable reporter activity (Fig. 6A), consistent with the idea that vRNPs containing MxA escape mutations utilize the classical importin-α/β nuclear import pathway and do not use alternative pathways. We confirmed this using immunofluorescence microscopy, where LMH cells pretreated with cycloheximide were infected with wild-type SC35M and mutant SC35M_2x in the presence of either 4-HPR, 4-MPR or DMSO and stained for NP (Fig. 6B). Ninety minutes post infection vRNPs were enriched in the nucleus of wild-type but not SC35M_2x-infected cells treated with DMSO or inactive 4-MPR, whereas treatment with 4-HPR led to retention of wild-type NP in the cytoplasm. None of the compounds had any effect on NP localization of SC35M_2x (Fig. 6B). Taken together, these results suggest that MxA escape mutations do not enable alternative importin-independent pathways.

## Discussion

AIVs can infect human individuals but rarely manage to establish a new virus lineage in the human population. A formidable barrier preventing sustained trans-species transmissions consists in the cell-autonomous innate immune defenses of the mammalian host. In humans, the IFN-inducible MxA protein is a major innate immunity factor that needs to be overcome by AIVs^21^. We previously identified adaptive mutations in the 1918 and 2009 pandemic viruses that allowed escape from MxA restriction and further showed that the escape mutations are a hallmark of all influenza A virus strains circulating in humans^33^. MxA escape could be assigned to three amino acids that form a distinct cluster in the body domain of NP. These residues are present in almost all circulating human influenza A virus, yet absent in AIVs. Here we demonstrate that introduction of the MxA escape mutations 100I/V, 283P, and 313Y into avian H5N1 and H7N7 viruses resulted in severe growth defects. We further show that the mutant H5N1 viruses readily acquired additional mutations in NP that secured genetic stability during passage in cells. Rescued viruses continued to exhibit impaired growth in almost all cell lines tested, irrespective of whether the cells were of mammalian or avian origin. Surprisingly, all of the stabilizing mutations in NP caused a loss of MxA escape and restored MxA sensitivity to the level of wild-type virus, except substitution G16D. These findings suggest that it is almost impossible for AIVs to gain MxA escape while maintaining viral fitness. Escape from MxA restriction and robust viral replication appear to be mutually exclusive without prior acquisition of a stabilizing mutation in NP such as 16D. This dichotomy might explain in part why AIVs rarely become pandemic.

While the exact mechanism by which the stabilizing NP mutations restore sensitivity to the antiviral action of MxA remains to be investigated, we have elucidated how MxA escape mutations cause viral attenuation. We found that the unexpected fitness loss is due to impaired transport of incoming vRNPs into the cell nucleus as shown by immunofluorescence analysis. Defective nuclear import was most prominent in avian LMH cells after infection with KAN-1_2x virus carrying the two NP mutations 100I and 313Y that are associated with MxA escape (Fig. 5A,B). Consistent with inefficient nuclear accumulation of vRNPs, primary transcript levels in KAN-1_2x infected LMH cells were also greatly reduced. Low level primary transcription could have been due to inefficient polymerase activity, however this possibility is highly unlikely since the NP carrying the two MxA escape mutations supported the H5N1 polymerase with comparable efficiency to wild-type NP in reconstitution assays (supplementary Figure S3). Additional evidence for inefficient nuclear vRNP accumulation was obtained in a VLP-based assay. It relied on H5N1 VLPs containing a reporter minigenome. Only primary transcription is performed in cells infected with such VLPs since the viral proteins required for secondary transcription and replication are absent. Therefore, reporter activity directly correlates with primary transcript levels and can be used as an indirect read-out for nuclear vRNP import efficiencies. Indeed, reporter activities were significantly lower in LMH cells when VLPs containing mutant NP were used instead of wild-type, consistent with defective transport of mutant vRNPs into the nucleus of LMH cells. In VLP-infected DF-1 cells, as well as mammalian A549 or MDCK cells, reporter activities in the presence of mutant and wild-type NP were comparable. This is expected, since viral growth of mutant KAN-1_2x and wild-type KAN-1 differed only slightly in these cell lines and primary transcription (Fig. 5A) as well as nuclear accumulation of vRNPs (data not shown) both occured with similar efficiency in DF1 cells.

Direct proof that a comparable block in nuclear vRNP import occurs when NP contains all three MxA escape mutations (100 V, 283P and 313Y) cannot be tested experimentally because the relevant H5N1 viruses are highly attenuated, with resultant selection for additional mutations. However, the H5N1 VLP-based infection assay revealed that viruses containing all three major MxA escape mutations led to significantly reduced reporter activity in all cell lines tested, highlighting a cell-type independent effect (Fig. 5C). Since these three amino acids do not interfere but rather enhance the H5N1 polymerase activity, the reduced reporter activity observed most likely mirrors impaired nuclear vRNP import.

We were able to show that the impairment of nuclear import of incoming vRNPs carrying MxA escape mutations occurs after fusion of the viral membrane with the endosomal membrane and most likely after vRNP release into the cytosol. Which subsequent steps in vRNP transport are affected is an open question that remains to be addressed. Nuclear import of incoming vRNPs is thought to be mediated by importin-α/β and relies largely on recognition of nuclear localization sites 1 and 2 (NLS1 and NLS2) of NP^38^,^39^,^42^.

That two different inhibitors of importin-α/β-dependent nuclear import of viral proteins, ivermectin and 4-HPR^46^,^47^,^48^ inhibit nuclear accumulation of vRNPs carrying MxA escape mutations, is compatible with the view that the importin-α/β nuclear import pathway is clearly involved. It is conceivable that mutations in the Mx patch of NP may decrease binding to either certain importin-α/β heterodimers, or to some other as yet unknown, host factors required for regulating efficient vRNP nuclear import. We favor the latter possibility because the severity of the vRNP import block differed significantly between avian DF1 and avian LMH cells although both cell types express comparable mRNA levels of all known importin-α and -β isoforms (data not shown). Moreover, the localization of the amino acids on NP associated with MxA escape does not correlate with either NLS1 or NLS2, making a direct interference with recognition of the NLS sites by importin-α isoforms unlikely. What is striking, however, is that mutations which allow evasion from an antiviral factor known to inhibit intracellular trafficking of distinct viral components can result directly in greatly reduced vRNP nuclear import efficiency, supporting the idea that this remains a step of influenza virus infection of key importance, with therapeutic implications^49^. The additional mutations in NP that were acquired by H5N1 strain KAN-1_3x during cell passage partly increase viral fitness, as well as nuclear vRNP import. Intriguingly, with the exception of one mutation (G16D), the stabilizing mutations restored MxA sensitivity to the level of wild-type virus by unknown mechanism(s). With the exception of G16D, the additional mutations are not found in human circulating strains, as expected^6^,^33^.

**Figure 7:**
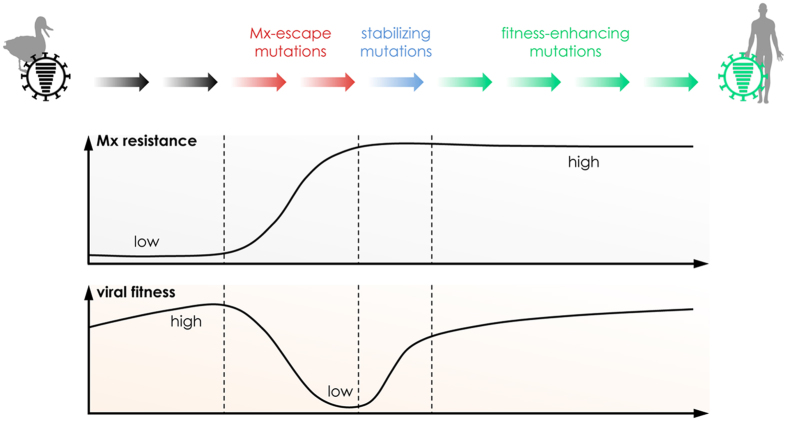
Model depicting the evolutionary bottleneck for avian influenza viruses encountering the host restriction factor MxA. To establish a new lineage in the human population, avian influenza A viruses have to acquire several adaptive mutations in almost all viral proteins, including MxA escape mutations in NP. However, the acquisition of MxA escape amino acids in NP is associated with severely reduced viral fitness, due to impaired nuclear import of vRNPs. Stabilizing mutations in NP (e.g. 16D) are required to overcome this fitness restriction, but are not sufficient to restore viral growth properties. As a consequence further additional mutations in NP and probably other viral gene products are required.

Our studies suggest that acquisition of MxA escape mutations is a double-edged sword for AIVs. To establish a new lineage in the human population AIVs must overcome not only MxA restriction but also the inherently associated attenuation (Fig. 7). Escape from Mx reduces the ability of the virus to carry out an essential function (nuclear import). Since the molecular signatures required on the surface of NP to allow nuclear import in human cells must always be the same, this provides an evolutionary bottleneck requiring convergent evolution on the part of all AIV attempting to jump to humans. This difficulty may explain why all 1918-descendent viruses maintained the NP carrying the original MxA escape patch. Of note, the pandemic 2009 H1N1 virus evolved a new but overlapping MxA escape patch, which was presumably partially acquired in swine under the pressure of porcine Mx proteins^33^. Chicken and duck Mx proteins have no restricting activity against influenza A viruses and selection for MxA escape may not occur in avian species^26^,^27^. In nearly 20 years since the first human infection with an H5N1 virus was reported, no MxA escape virus of the H5N1 subtype has emerged, highlighting the difficulty of acquiring MxA escape mutations required for circulation in the human population. A potential threat could arise by reassortment events between H5N1 and circulating human strains. However, H5N1 reassortant viruses containing either the pandemic 2009 H1N1 NP^30^ or the 1918 NP (data not shown) showed a degree of MxA escape but decreased viral fitness. This suggests that attenuation associated with MxA escape is a complex trait that is influenced not only by NP but likewise by other viral proteins. As exemplified by SC35M_3x + 16D, acquisition of MxA escape mutations is possible without impaired viral fitness in mammalian cells. Similar to circulating human influenza viruses, SC35M contains diverse mammalian-adaptive mutations in different viral segments due to extensive passaging in mice^36^, which probably compensate attenuation mediated by the MxA escape mutations (Fig. 7). Based on these results, it is tempting to speculate that H5N1 viruses have a low pandemic potential. This could be different for other AIVs. We recently found that the single amino acid 52N found in the NP gene of an H7N9 strain (originally acquired by gene reassortment from an avian H9N2 virus)^50^ was sufficient to confer minor MxA escape without obvious loss of viral fitness^51^. Of note, this amino acid was not found in human H5N1 isolates nor in circulating H5N1 strains, further highlighting the difficulty of H5N1 viruses in evading MxA antiviral activity.

## Material and Methods

### Cells and viruses

Madin-Darby canine kidney II (MDCK II) were kindly provided by Georg Kochs, University of Freiburg, Germany. Human alveolar basal epithelial cells (A549), human embryonic kidney cells (293T), chicken hepatocellular carcinoma cells (LMH), and chicken fibroblast (DF1) cells were obtained from the American Type Culture Collection (ATTC). A549 cells constitutively expressing MxA (A549-MxA) or expressing MxA-specific shRNAs (A549-shMxA) were kindly provided by Richard E. Randall, University of St Andrews, St Andrews, UK^29^. Recombinant viruses A/Thailand/1(KAN-1)/04 (H5N1), A/SC35M (H7N7), and mutant variants of both viruses were generated by reverse genetics as described^33^. Cells were seeded in 6-well plates and infected at the indicated multiplicities of infection (MOI). Virus titers were determined by plaque assays.

### Compounds

Cycloheximide and ivermectin (Sigma Aldrich), N-(4-hydroxyphenyl) retinamide (4-HPR) (Tocris Biosciences), N-(4-methoxyphenyl) retinamide (4-MPR) (Toronto Research Chemicals), octadecyl rhodamine B chloride (R18) and 3,3′-dioctadecyl-5,5′-di(4-sulfophenyl)-oxacarbocyanine (SP-DiOC18) (Molecular Probes).

### Sequencing

Supernatant (500 μl) from infected cells was mixed with 1 ml of TRIzol^®^ to isolate total RNA according to the manufacturer’s protocol (Invitrogen). cDNA synthesis and DNA amplification was performed using OneStep RT-PCR-Kit (Qiagen) and segment-specific primers. Deep sequencing was performed as described in^52^.

### Plasmid construction

pHW2000 rescue plasmids^53^ were used for site directed mutagenesis. The mutated ORF were cloned into pCAGGS expression plasmids using restriction enzymes NotI and XhoI (Fermentas).

### Immunofluorescence

Cells were seeded on glass coverslips. One hour prior to infection, cells were pretreated with 100 μM cycloheximide and subsequently infected at an MOI of 50 in the presence of 100 μM cycloheximide on ice for 40 minutes. Virus particles were allowed to enter the cells at 37 °C for 5 minutes. Infected cells were washed and further incubated at 37 °C in the presence of 100 μM cycloheximide. After indicated time points, supernatant was removed and cells were fixed with 4% PFA. Images were obtained by confocal microscopy at 63× magnification.

### Primer extension analysis

Viral transcript levels in infected cells were determined by primer extension analysis using specific primers for the NA segment (mRNA, cRNA, and vRNA) and for cellular 5 S RNA, as described^54^. For primary transcription analysis, cells were pretreated with 100 μM cycloheximide for 1 h before infection at an MOI of 50. At specific time points cells were collected in TRIzol^®^ and RNA was isolated according to the manufacturer’s protocol (Invitrogen).

### Virus-like particle (VLP) assay

293T cells (producer cells) were transiently transfected with a mixture of expression plasmids coding for PB2, PB1, PA, HA, NA, M2, M1, and NEP, as well as wild-type or mutant NP proteins of KAN-1. In addition, a polymerase I (Pol I)-driven viral minigenome construct expressing the reporter protein firefly luciferase was cotransfected together with a plasmid that constitutively expresses renilla luciferase and served to normalize transfection efficiency. 1000 ng of each plasmid was used, except for pCAGGS-PA and -M2 (100 ng) and of pCAGGS-M1 (2000 ng). 48 hours post transfection supernatant was collected, centrifuged at 1000 rpm for 10 minutes and analyzed by Western blotting to assess VLP production efficiency of the different NP proteins. The producer cells were lysed and firefly and renilla luciferase activities were measured to determine polymerase activities of the different NP variants. Finally, the supernatant of the donor cells was used for infection of indicator cells, which were lysed after 10 h to determine luciferase activities as described.

### Polymerase reconstitution assay

Polymerase activity was analyzed using the polymerase reconstitution (minireplicon) assay described previously^33^. Briefly, HEK293T cells were transiently transfected with pCAGGS expression plasmids coding for all polymerase subunits, the indicated NP variants, and a (Pol I)-driven viral minigenome construct expressing the reporter protein firefly luciferase. A plasmid constitutively expressing renilla luciferase served to normalize transfection efficiency. Firefly luciferase activity was normalized to renilla luciferase activity and served as an indicator for polymerase activity. To evaluate the antiviral effect of MxA, an MxA-encoding expression plasmid was cotransfected. The inactive mutant MxA_T103A served as control in parallel experiments. Polymerase activity in the presence of the inactive MxA_T103A was used to normalize polymerase activity in the presence of MxA (relative activity). Polymerase reconstitution in avian cells was performed as described above with the exception that minigenome RNA was expressed under the control of a chicken Pol I promotor^54^.

### siRNA knockdown

The expression of respective genes was silenced by reverse siRNA transfection using 5 μl Lipofectamine RNAiMAX (Thermo Fisher) per 6-well. The transfection reagent was diluted in 625 μl Opti-MEM (Thermo Fisher) and incubated for 10 min at room temperature. The siRNA was diluted in an additional 625 μl Opti-MEM. The two solutions were mixed, incubated for 15 min and transferred into a 6-well (1250 μl). A549-MxA cells (2*10^5^) in a volume of 1250 μl DMEM containing 20% FCS (without antibiotics) were seeded on top, resulting in a final siRNA concentration of 30 nM (final volume 2500 μl). 72 h post transfection cells were infected. The MxA-targeting siRNA was purchased from Qiagen while the non-targeting control siRNA was bought from Dharmacon.

### Fusion assay

Fusion of viral and endosomal membranes was visualized according to a protocol described elsewhere^55^. Briefly, wild-type and mutant SC35M viruses were labeled with two fluorescent dyes, octadecyl rhodamine B chloride (R18) and 3,3′-dioctadecyl-5,5′-di(4-sulfophenyl)-oxacarbocyanine (SP-DiOC18), in a ratio of 1:2 with final concentrations of 22 μM for R18 and 46 μM for SP-DiOC18. Each virus stock was incubated with both dyes for 1 hour in the dark. Labeled virus was filtered using a 0.22 μm pore filter. Viruses were either stored at −80 °C or directly used for infection experiments. Cells on cover slips were infected on ice with the labeled viruses at an MOI of 100. After 30 minutes, cells were washed and incubated at 37 °C for indicated time points. Images were taken using a Zeiss confocal microscope.

## Additional Information

**How to cite this article**: Götz, V. *et al.* Inf luenza A viruses escape from MxA restriction at the expense of efficient nuclear vRNP import. *Sci. Rep.* **6**, 23138; doi: 10.1038/srep23138 (2016).

## Supplementary Material

Supplementary Information
