# Corrigendum: Influenza A viruses escape from MxA restriction at the expense of efficient nuclear vRNP import

**DOI:** 10.1038/srep25428

**Published:** 2016-05-09

**Authors:** Veronika Götz, Linda Magar, Dominik Dornfeld, Sebastian Giese, Anne Pohlmann, Dirk Höper, Byung-Whi Kong, David A. Jans, Martin Beer, Otto Haller, Martin Schwemmle

Scientific Reports 6: Article number: 23138;10.1038/srep23138 Published online: 03182016; Updated: 05092016

The Acknowledgements section in this Article is incomplete.

“We thank Richard Randall for providing A549 cells stably expressing MxA or shMxA, Georg Kochs for providing the NP-specific antibody and Geoffrey Chase, Georg Kochs and Peter Staeheli for critically reading of the manuscript.”

should read:

“We thank Richard Randall for providing A549 cells stably expressing MxA or shMxA, Georg Kochs for providing the NP-specific antibody and Geoffrey Chase, Georg Kochs and Peter Staeheli for critically reading of the manuscript. This was work was supported by the Deutsche Forschungsgemeinschaft (SCHW 632/15 and SFB 1160, project 13) to MS.”

