# Supplementary material for: Influenza A viruses escape from MxA restriction at the expense of efficient nuclear vRNP import
Source: Sci Rep. 2016 Mar 18;6:23138. doi: 10.1038/srep23138 (PMC4796820; doi:10.1038/srep23138)
Supplement: Supplementary Information [file srep23138-s1.pdf]

Influenza A viruses escape from MxA restriction at the expense of efficient nuclear vRNP import

Veronika Götz[1], Linda Magar[1], Dominik Dornfeld[1], Sebastian Giese[1], Anne Pohlmann[2], Dirk Höper[2], Byung-Whi Kong[3], David A. Jans[4], Martin Beer[2], Otto Haller[1] and Martin Schwemmle[1*]

[1]Institute of Virology, University Medical Center Freiburg, D-79104 Freiburg, Germany

[2]Institute of Diagnostic Virology, Friedrich-Loeffler-Institute, 17493 Greifswald-Insel Riems, Germany

[3]Center of Excellence for Poultry Science, University of Arkansas Fayetteville, AR 72701, USA

[4]Nuclear Signaling Laboratory, Department of Biochemistry and Molecular Biology, Monash University, Melbourne, Victoria, Australia

*Corresponding author:

E-mail: martin.schwemmle@uniklinik-freiburg.de (MS)

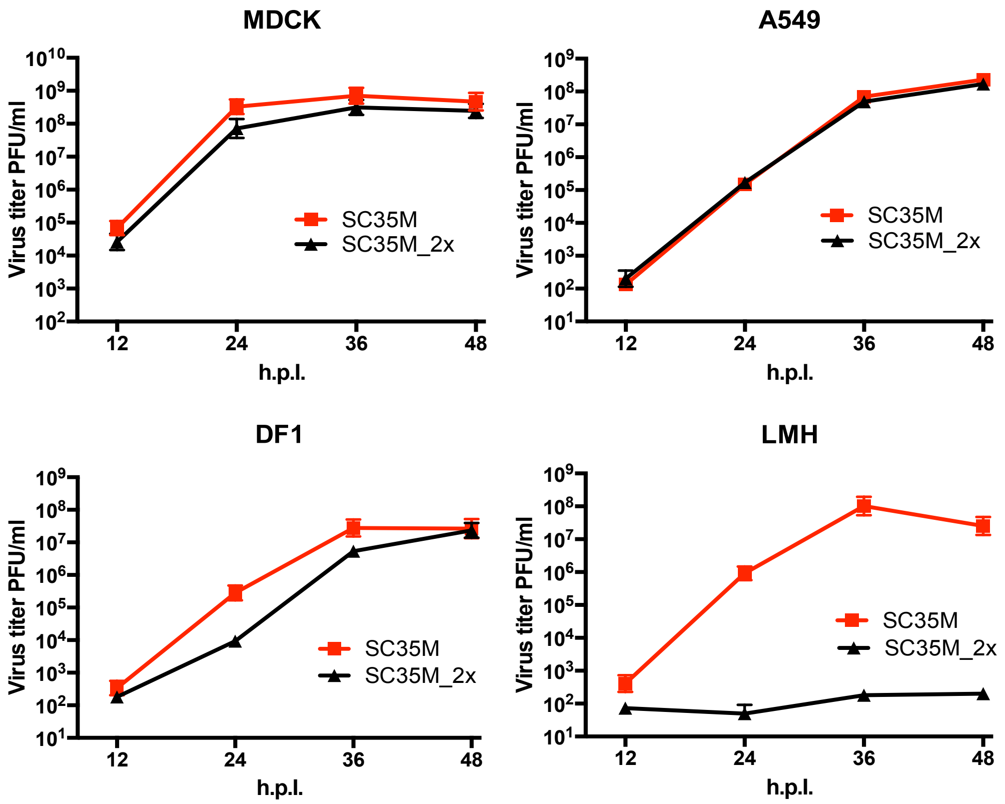

Figure S1. Recombinant SC35M viruses carrying MxA escape mutations in NP are attenuated to varying degrees in different cell culture systems.
Cells were infected at an MOI of 0.001 of wild-type SC35M (SC35M) or SC35M_2x. At the indicated time points post infection (p.i.), virus titers were determined by plaque assay. Error bars indicate the standard deviation of the mean of at least three independent experiments.

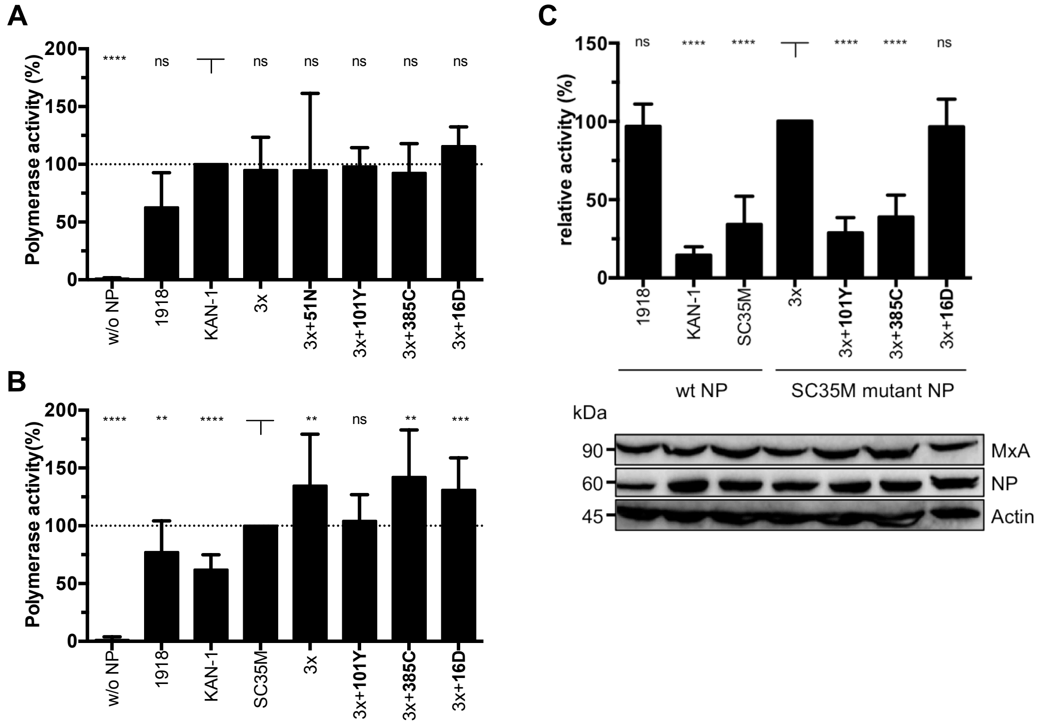

Figure S2. Effect of the additional adaptive mutations on polymerase activity of KAN-1_3x or SC35M_3x in human cells.

A and B) Polymerase activity in the presence of antivirally inactive MxA_T103A. HEK293T cells were transiently transfected with expression plasmids coding for PB2, PB1, PA of KAN-1 (A) or SC35M (B), wild-type NP (1918; KAN-1; SC35M) or the indicated mutant proteins of KAN-1 (A) or SC35M (B), a minigenome encoding the firefly luciferase and a renilla luciferase expression plasmid to normalize for variations in expression efficiency. NP was omitted as a negative control (w/o NP). Error bars indicate the standard deviation from the mean of at least 3 independent experiments. Student´s T test was performed to determine the P value **P<0.01, ***P<0.001, ****P<0.0001, not significant (ns).

C) Polymerase activity in the presence of MxA. Polymerase reconstitution was carried out as in (B). Polymerase activity in the presence of antivirally inactive MxA_T103A was used to normalize the data obtained with MxA (relative activity). Western blot analysis was performed to determine the expression levels of NP and MxA. Error bars indicate the standard deviation of the mean of at least 3 independent experiments. Student´s T test was performed to determine the P value ****P<0.0001, not significant (ns).

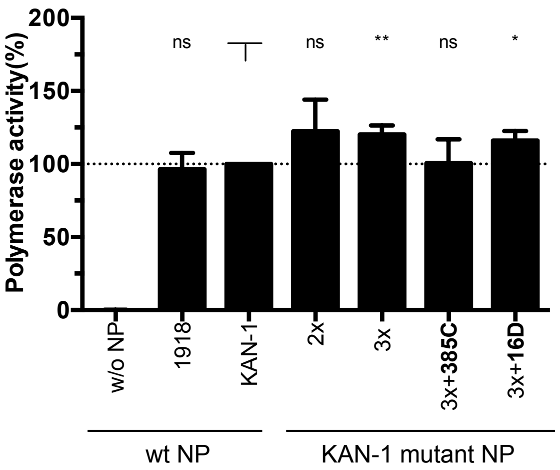

Figure S3. Polymerase activity with KAN-1 NP mutants in avian LMH cells.
LMH cells were transiently transfected with expression plasmids coding for PB2, PB1, PA of KAN-1, wild-type NP (1918; KAN-1) or the indicated KAN-1 mutant proteins, a minigenome encoding the firefly luciferase and a renilla luciferase expression plasmid to normalize for variations in expression efficiency. Error bars indicate the standard deviation from the mean of at least 3 independent experiments. Student´s T test was performed to determine the P value *P<0.05, **P<0.01, not significant (ns).

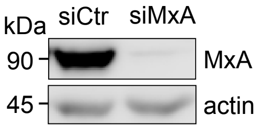

Figure S4. si RNA knockdown efficiency of A549-MxA cells.
A549-MxA cells, constitutively expressing MxA, were either treated with an siRNA targeting MxA (siMxA) or a non-targeting control siRNA (siCtr). Western blot analysis was performed to evaluate knockdown efficiency.

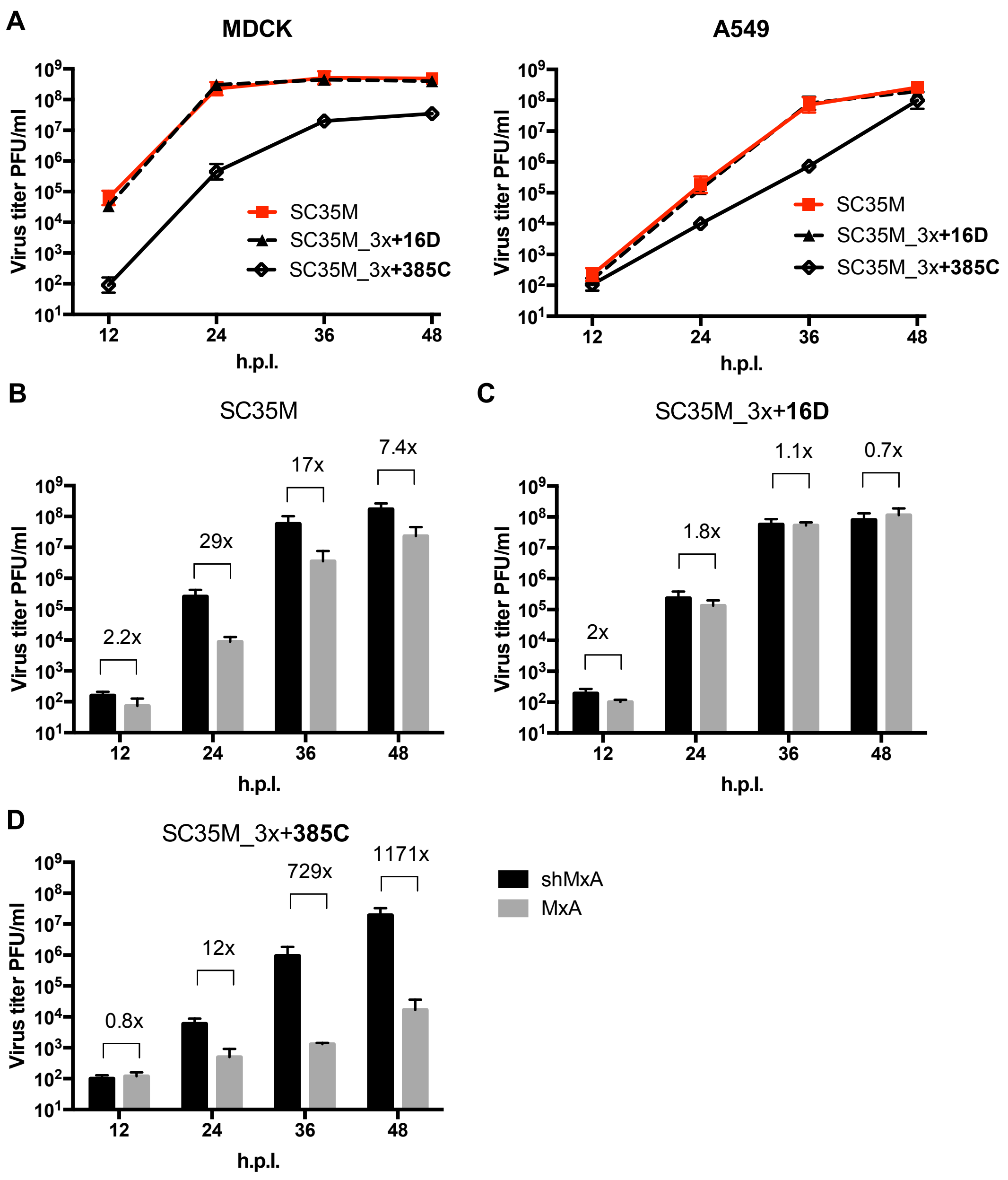

Figure S5. Recombinant SC35M_3x mutants harboring stabilizing mutations show varying degrees of attenuation and MxA escape.

A) Cells were infected at an MOI of 0.001 with wild-type SC35M (SC35M) or the indicated mutant viruses. At the indicated time points post infection (p.i.), virus titers were determined by plaque assay. Error bars indicate the standard deviation of the mean of at least three independent experiments.

B-D) A459 cells expressing (MxA) or lacking MxA (shMxA) were infected at an MOI of 0.001 of wild-type SC35M (SC35M) or the indicated mutant viruses. At the indicated time points post infection (p.i.), virus titers were determined by plaque assay. Error bars indicate the standard deviation of the mean of 3 independent experiments. Fold differences in virus titers are indicated.

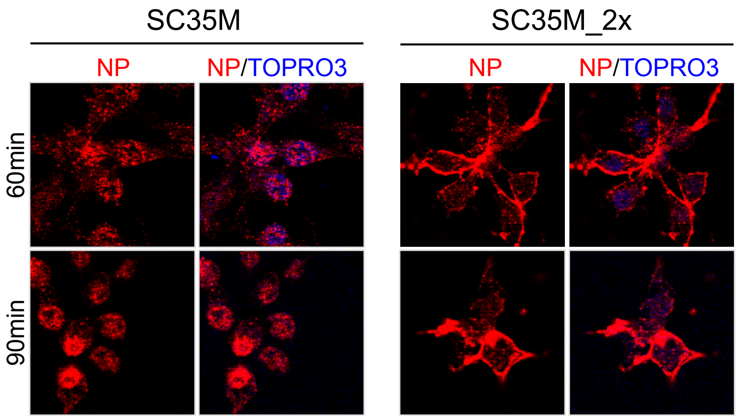

Figure S6. Impaired nuclear import of incoming vRNPs of SC35M carrying MxA escape mutations.
Detection of incoming vRNPs in LMH cells infected with wild-type SC35M or mutant SC35M_2x at an MOI of 50 in the presence of 100µg/ml cycloheximide. Infection was carried out on ice for 40 minutes to synchronize virus entry and further incubated at 37°C for 60 or 90 minutes. Incoming vRNPs were visualized using a NP-specific antibody. Cell nuclei were visualized by TOPRO3 staining.

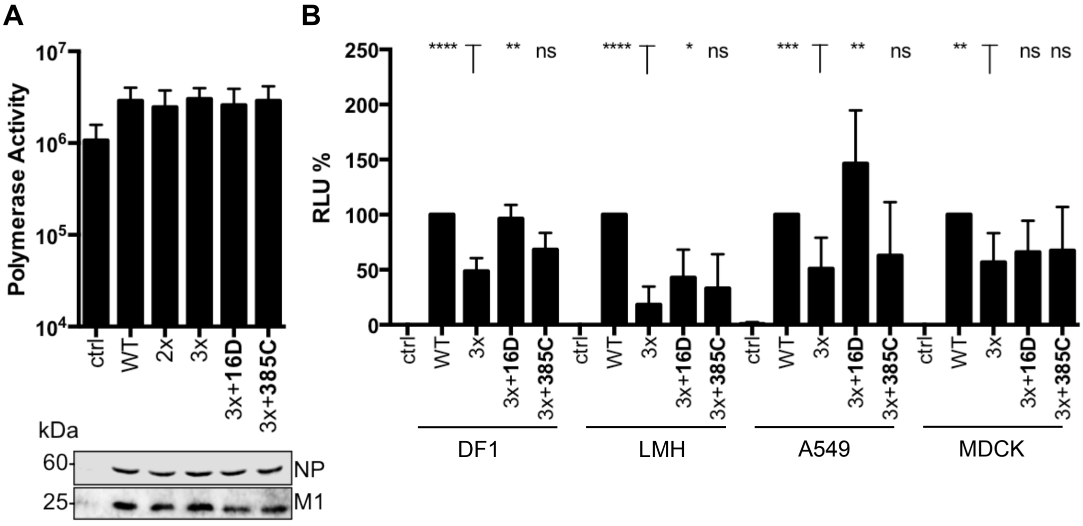

Figure S7. Additional mutations in NP_3x partially restore VLP activity indicating improved nuclear vRNP import.

To generate VLP, HEK293T cells were transiently transfected with expression plasmids coding for all viral proteins of KAN-1 including wild-type (WT) or the indicated mutant NP proteins, a minigenome coding for firefly luciferase and a renilla luciferase expression plasmid to normalize for variations in expression efficiency. 48 hours post transfection cell supernatant containing the VLPs was collected. (**A**) Viral polymerase activity was determined in the producer HEK293T cells. Error bars indicate the standard deviation from the mean of at least 3 independent experiments. Levels of NP and M1 were determined by Western blot analysis in the cell supernatants to control for equal VLP production.

B) VLPs were used to infect the indicated cell lines and luciferase expression was determined 10 hours post infection. Error bars indicate the standard deviation from the mean of at least 4 independent experiments. Student´s T test was performed to determine the P value *P<0.05, **P<0.01, ***P<0.001, ****P<0.0001, not significant (ns).

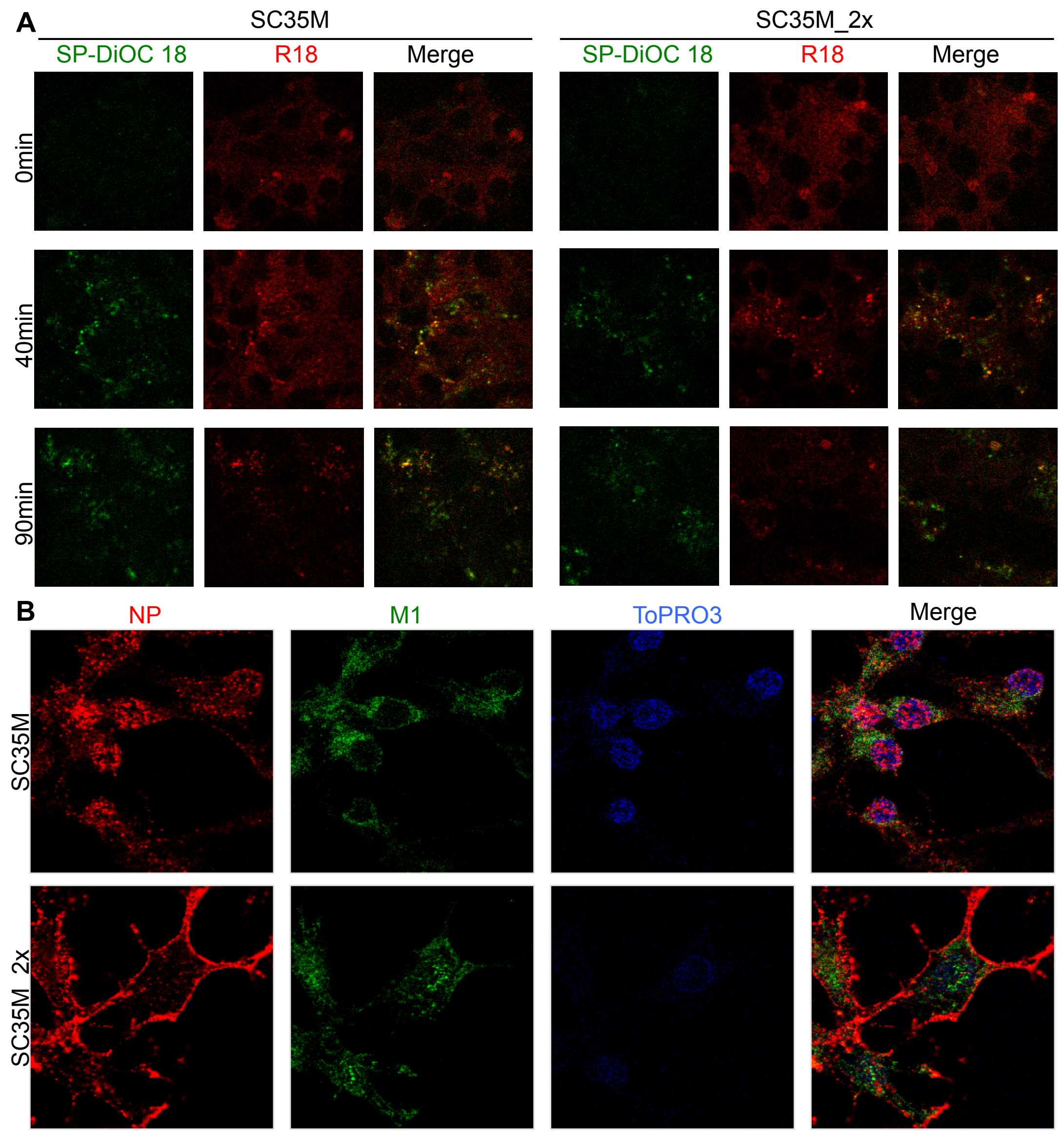

Figure S8. Fusion and uncoating is not affected in cells infected with SC35M_2x.
A) Fusion of viral and endosomal membranes was visualized using wild-type SC35M or mutant SC35M_2x labeled with two fluorescent dyes (R18 and SP-DiOC18). Infection of LMH cells was carried out on ice for 40 minutes to synchronize virus entry and further incubated at 37°C. After the indicated minutes, cells were fixed and analyzed by confocal microscopy. Non-fused viruses are red (R18) due to FRET and self-quenching of DiOC 18. Upon fusion green signal appears.
B) To determine uncoating events, LHM cells were infected with wild-type SC35M or mutant SC35M_2x at an MOI of 50 in the presence of 100µg/ml cycloheximide. Infection was carried out on ice for 40 minutes to synchronize virus entry and further incubated at 37°C. After 90 minutes, cells were fixed and the viral nucleoprotein and M1 were stained.

Virus rescue	Virus stock	MDCK	A549	A549-MxA	DF1	LMH
**KAN-1_2x #1**<br>Stock titer: $3x10^8$PFU/ml						
NP_100I, 313Y	yes	yes	yes	yes	yes	yes
other mutations in NP	no	no	no	no	no	no
**KAN-1_3x #1**<br>Stock titer: $3x10^3$PFU/ml						
NP_100V, 283P, 313Y	yes	yes	n.d	yes	n.d	yes
other mutations in NP	no	G16D	n.d	G16D	n.d.	D101Y
**KAN-1_3x #2**<br>Stock titer: $1x10^4$PFU/ml						
NP_100V, 283P, 313Y	yes	yes	yes	n.d	yes	yes
other mutations in NP	n.d.	Y385C	Y385C	n.d	Y385C	Y385C,<br>C101Y
**KAN-1_3x #3**<br>Stock titer: $2x10^7$PFU/ml						
NP_100V, 283P, 313Y	yes					
other mutations in NP	D51N					

Supplementary Table S1

Virus rescue	Virus stock	MDCK	A549-MxA	LMH
**KAN-1_3x #1**<br>Stock titer: 3x10^{3}PFU/ml				
NP_100V, 283P, 313Y	yes	yes	yes	yes
NP	no	G16D (99%)	G16D (99%)	D101Y (97%)
PB2	S635A (21%)	-	-	-
PB1	L10V (99%)	L10V (99%)	L10V (97%)	L10V (99%)
HA				D474E (35%)<br>N475K (17%)<br>A476T (17%)<br>A476I (35%)
NA	S35G (11%)	-	-	-
**KAN-1_3x #2**<br>Stock titer: 1x10^{4}PFU/ml				
NP_100V, 283P, 313Y	n.d	yes	n.d	yes
NP	n.d.	Y385C (95%)	n.d.	D101Y (72%)<br>Y385C (22%)
PB2		M47T (24%)<br>M47I (25%)<br>W49G (19%)<br>W49C (19%)		M47T (30%)<br>M47I (29%)<br>W49G (22%)<br>W49C (20%)

Supplementary Table S2
